# Probiotic intervention improves cholestatic parameters in patients with drug-induced liver injury: a real-world retrospective study

**DOI:** 10.3389/fnut.2025.1657629

**Published:** 2025-09-26

**Authors:** Na Li, Penghui Liu, Jiwu Guo, Lingyun Guo, Jizhen Wang, Ziyuan Mou, Jie Mao

**Affiliations:** The General Surgery Department, Lanzhou University Second Hospital, Lanzhou, China

**Keywords:** drug-induced liver injury, probiotic intervention, liver function, cholestatic, therapeutic efficacy

## Abstract

**Objective:**

This study aimed to investigate the impact of gut microbiota intervention on the therapeutic outcomes in patients with drug-induced liver injury (DILI).

**Methods:**

A total of 120 patients with DILI who were hospitalized at the Second Hospital of Lanzhou University from January 2023 to December 2024 were retrospectively enrolled. Patients were divided into an intervention group and a control group based on whether they received gut microbiota intervention therapy. Quantitative data were presented as median and interquartile range, and the Mann–Whitney U test was used for intergroup comparisons. Categorical variables were compared between groups using chi-square tests. Logistic regression analysis was used to identify factors associated with liver injury severity and to evaluate the effect of gut microbiota intervention.

**Results:**

After treatment, GGT, ALP, TBIL, DBIL and IBIL decreased significantly more in the intervention group than in the control group, and the differences were statistically significant (*p* < 0.01). Before treatment, the intervention group had a higher proportion of patients with grade 1 and grade 4 DILI, while the control group had more patients with grade 2 and grade 3 DILI. After treatment, the proportion of grade 1 patients in the intervention group increased significantly, whereas the proportion of grade 4 patients decreased markedly. These changes were statistically significant between the two groups (*p* < 0.01). Logistic regression analysis showed that the risk of liver injury was significantly reduced in the intervention group after treatment (OR = 0.018, *p* < 0.01). In terms of model prediction accuracy, the prediction rate for moderate liver injury was relatively high (91.0%), whereas the accuracy for acute liver failure and severe liver injury was lower. The overall prediction accuracy of the model was 75.0%. Pearson correlation analysis revealed that post-treatment DILI grade was significantly associated with treatment regimen, TBIL, ALP and GGT (*p* < 0.01).

**Conclusion:**

Probiotic intervention significantly improved cholestasis-related parameters and reduced the severity of liver injury in patients with DILI. This real-world study provides novel clinical evidence supporting microbiota-targeted therapy as a promising adjunctive strategy, particularly for cholestasis-predominant DILI.

## 1 Introduction

Drug-induced liver injury (DILI) refers to liver damage caused by medications, and its pathogenesis is complex, involving multiple mechanisms such as oxidative stress, mitochondrial dysfunction, metabolic disturbances, and immune-mediated inflammation. The severity of liver injury can vary widely, ranging from mild elevations in liver enzymes to serious outcomes such as acute liver failure (1, 2). Current treatment strategies primarily involve drug withdrawal and supportive care. However, a subset of patients still experience poor outcomes due to irreversible liver damage or progression to chronic liver injury (3). Therefore, exploring more effective intervention strategies to improve the prognosis of patients with DILI has become a growing focus of research.

In recent years, the role of the gut-liver axis in liver diseases has attracted widespread attention (4).

The gut microbiota can influence liver health through multiple mechanisms, including modulation of host immune system, detoxification of harmful metabolites, participation in bile acid metabolism, synthesis of short-chain fatty acids, and maintenance of intestinal barrier integrity. Dysbiosis of the gut microbiota is closely associated with various liver diseases, including metabolic dysfunction-associated steatotic liver disease (MASLD), alcoholic liver disease (ALD), viral hepatitis, and autoimmune liver diseases. It plays a critical role in immune regulation and the maintenance of hepatic homeostasis (5, 6). Microecological interventions such as probiotics and prebiotics have demonstrated great potential in modulating the gut microbiota and improving liver function. Mechanistically, probiotics can restore microbial balance, suppress intestinal permeability, reduce endotoxin translocation, downregulate proinflammatory cytokines, and regulate bile acid homeostasis (7, 8). Clinical and preclinical studies have reported that probiotics alleviate hepatic steatosis in MASLD, improve intestinal barrier function and inflammation in ALD, and exert immunomodulatory benefits in viral and autoimmune hepatitis. These findings provide a strong rationale for testing probiotics in other forms of liver injury (9, 10).

Although previous studies have examined the role of the gut microbiota in liver diseases, evidence supporting probiotic therapy in DILI is still scarce. Most investigations have focused on hepatocellular protection and immune modulation, with relatively few targeting the gut microbiota as a therapeutic pathway (11, 12). The effectiveness of probiotics in promoting liver function recovery and improving clinical outcomes in DILI, as well as the underlying mechanisms, remains unclear. Therefore, this study aimed to evaluate the impact of probiotic intervention on liver function recovery and patient outcomes in DILI, with the goal of providing novel therapeutic insights and expanding the clinical potential of microbiota-based therapies in liver injury.

## 2 Material and methods

### 2.1 Study subjects

This was a retrospective observational cohort study conducted at the Second Hospital of Lanzhou University. A total of 120 patients diagnosed with DILI between January 2023 and December 2024 were identified through electronic medical records. According to the actual treatment received during hospitalization, patients were classified into an intervention group (*n* = 40), who received conventional therapy plus probiotics, and a control group (*n* = 80), who received conventional therapy alone. No randomization was performed.

All patients met the diagnostic criteria for DILI as defined in the 2023 edition of the Chinese Guidelines for the Diagnosis and Treatment of DILI. Exclusion criteria were: (1) liver injury clearly attributable to non-drug-related causes; (2) severe uncontrolled comorbidities (e.g., cardiovascular, renal, or metabolic diseases); (3) documented allergy or intolerance to probiotic preparations; (4) incomplete clinical data or insufficient follow-up information; (5) participation in other clinical trials or receipt of additional investigational treatments that might affect liver function.

Written informed consent for the use of anonymized medical data was routinely obtained from all patients upon admission, in accordance with institutional regulations. The study protocol was reviewed and approved by the Ethics Committee of the Second Hospital of Lanzhou University, and all procedures conformed to the principles of the Declaration of Helsinki.

### 2.2 Data collection

The study collected clinical data including patients’ demographic information, grading of liver injury severity, liver function indicators, and clinical regression indicators. Liver function indicators included alanine aminotransferase (ALT), aspartate aminotransferase (AST), gamma-glutamyl transferase (GGT), alkaline phosphatase (ALP), total protein (TP), albumin (ALB), globulin (GLB), albumin-to-globulin ratio (A/G), total bilirubin (TBIL), direct bilirubin (DBIL), and indirect bilirubin (IBIL). Clinical regression indicators included intensive care unit (ICU) treatment, artificial liver treatment, and length of hospitalization. Relevant data were obtained through the hospital’s electronic medical record system.

Drugs most likely to cause DILI can be categorized into five groups: Antibiotics; Antituberculosis drugs (isoniazid, rifampin, pyrazinamide, and/or ethambutol); Antineoplastic agents (chemotherapy drugs such as capecitabine, targeted therapies such as sorafenib or apatinib); Traditional Chinese herbs/medicines (e.g., *Polygonum multiflorum*, compound toad venom, Yangzheng compound); and other drugs (including nonsteroidal anti-inflammatory drugs, statins, and anticonvulsants). The distribution of each causative drug was assessed in both the probiotic and control groups. Due to sample size limitations and the study’s primary focus on the probiotic intervention effect, detailed subgroup analyses at the individual drug level were not conducted.

According to the 2023 Chinese Guidelines for the Diagnosis and Treatment of DILI, remission was defined as the period during hospitalization when liver function tests returned to normal or near-normal levels, the severity grade of liver injury decreased, and clinical symptoms improved.

### 2.3 Severity grading criteria

According to the 2023 Chinese Guidelines for the Diagnosis and Treatment of DILI, severity is graded as follows: Grade 0, no liver injury; Grade 1, mild; Grade 2, moderate; Grade 3, severe; Grade 4, acute liver failure; Grade 5, fatal. These criteria are widely adopted in China but differ slightly from Western definitions, hence we provide this overview for international readers.

### 2.4 Treatment regimen

All patients received supportive therapy according to the standard practice of our hospital. This included magnesium isoglycyrrhizinate (MgIG) and amino acids, which are commonly used hepatoprotective agents in China. MgIG is recommended in the 2023 Chinese Guidelines for the Diagnosis and Treatment of DILI (Grade 1A) for patients with hepatocellular or mixed injury. Liver hydrolysate, although not guideline-endorsed, has been occasionally used in our center as an empirical supportive agent to provide amino acids and peptides for hepatocyte repair. Both groups received the same background therapy to ensure comparability, and the only difference between groups was the addition of probiotics in the intervention group. In our hospital, probiotics (Bifidobacterium quadruple viable tablets) have occasionally been prescribed off-label as an adjunctive therapy in patients with DILI, particularly when gut dysbiosis or cholestatic manifestations were considered clinically relevant. This reflects real-world clinical practice rather than a study-driven intervention.

Control Group: conventional treatment (MgIG + amino acids + liver hydrolysate).

Intervention Group: conventional therapy plus probiotic intervention. Probiotics consisted of Bifidobacterium quadruple viable tablets (Trade name: Shiliankang, National Drug Approval Number: S20060010; Hangzhou Yuanda Biopharmaceutical Co., Ltd.), administered orally at a dose of 0.21 g per tablet, 3 tablets per dose, 3 times daily. The probiotic regimen was continued until liver function improved or the patient was discharged.

### 2.5 Statistical analysis

Statistical analysis were conducted using SPSS 26.0. Quantitative data were expressed as the median (M) and interquartile range (IQR, P25-P75). Changes in liver function = post-treatment value—pre-treatment value. The Mann–Whitney U test was used for intergroup comparisons. Categorical variables were compared between groups using chi-square tests. Statistical significance was defined as *p* < 0.05. Logistic regression analysis was conducted to identify factors associated with liver function recovery and to establish a predictive model evaluating the impact of gut microbiota intervention on liver injury outcomes.

## 3 Results

### 3.1 Comparison of baseline characteristics

The causative drugs for DILI were categorized into five groups: antibiotics, anti-tuberculosis drugs, antineoplastic agents, traditional Chinese herbs/medicines, and others. The distribution of these categories was assessed between the probiotic and control groups. Antibiotics and anti-tuberculosis agents were the most frequent culprits, followed by antineoplastic agents and herbal medicines. The overall distribution of causative drug categories showed no significant difference between the two groups (χ^2^ = 0.39, *p* = 0.98) (Table 1).

**Table 1:** Distribution of causative drugs between groups.

Causative drug category	Control group (*n* = 80)	Intervention group (*n* = 40)
Antibiotics [*n* (%)]	20 (25.0)	12 (30.0)
Anti-tuberculosis drugs [*n* (%)]	22 (27.5)	10 (25.0)
Antineoplastic agents [n (%)]	18 (22.5)	8 (20.0)
Herbal [*n* (%)]	12 (15.0)	6 (15.0)
Others [*n* (%)]	8 (10.0)	4 (10.0)
Total [n (%)]	80 (100)	40 (100)

Variables are expressed as *n* (%); *N*, number.

Comparison of baseline characteristics showed that the median age of patients in the intervention group was 60.0 years, which was higher than that of 54.5 years in the control group (*p* < 0.01). In addition, INR values were slightly higher in the intervention group compared with the control group (median 1.10 vs. 1.05, *p* < 0.05), although both remained within the normal physiological range (0.8–1.2) and were far below the threshold typically used to define coagulopathy in severity grading (e.g., ≥1.5). No statistically significant differences were observed between the two groups in LDH, ALB, TP, height, weight, abdominal circumference, gender distribution, tobacco addiction, alcoholism, fatty liver, hepatitis, length of hospitalization, ICU treatment, or artificial liver treatment (all *p* > 0.05) (Table 2). Apart from probiotic administration, no significant differences in baseline treatment regimens were observed between the two groups.

**Table 2:** Baseline characteristics of the study population.

Indicators	Control group (*n* = 80)	Intervention group (*n* = 40)	χ^2^/U	*p*
Gender [*n* (%)]			0.067	0.796
Female	38 (47.50)	18 (45.00)		
Male	42 (52.50)	22 (55.00)		
Tobacco addiction [*n* (%)]			1.693	0.193
No	55 (68.75)	32 (80.00)		
Yes	25 (31.25)	8 (20.00)		
Alcoholism [*n* (%)]			0.108	0.743
No	64 (80.00)	33 (82.50)		
Yes	16 (20.00)	7 (17.50)		
Fatty liver [*n* (%)]			0.313	0.576
No	54 (67.50)	29 (72.50)		
Yes	26 (32.50)	11 (27.50)		
Hepatitis [*n* (%)]			1.678	0.195
No	40 (50.00)	25 (62.50)		
Yes	40 (50.00)	15 (37.50)		
ICU treatment [*n* (%)]			2.026	0.155
No	39 (48.75)	25 (62.50)		
Yes	41 (51.25)	15 (37.50)		
Artificial liver treatment [*n* (%)]			0.752	0.386
No	56 (70.00)	31 (77.50)		
Yes	24 (30.00)	9 (22.50)		
Age [M(P_25,_ P_75_)]	54.500 (45.3,62.8)	60.000 (56.3,66.8)	1082.000	0.004**
Height [M(P_25,_ P_75_)]	165.000 (160.0,172.0)	166.000 (159.3,173.0)	1489.500	0.538
Weight [M(P_25,_ P_75_)]	60.000 (52.3,70.0)	58.150 (53.2,66.6)	1511.000	0.620
Abdominal circumference [M(P_25,_ P_75_)]	81.700 (75.7,86.3)	77.850 (72.8,84.0)	1284.500	0.079
Length of hospitalization [M(P_25,_ P_75_)]	9.000 (7.0,13.0)	8.000 (5.0,15.5)	1386.000	0.232
LDH [M(P_25,_ P_75_)]	269.500 (202.5,396.5)	230.000 (191.3,369.0)	1355.000	0.173
ALB [M(P_25,_ P_75_)]	37.600 (32.8,41.8)	36.950 (32.2,39.5)	1414.000	0.300
TP [M(P25, P75)]	69.950 (64.8,75.6)	72.650 (66.1,77.1)	1422.000	0.322
INR [M(P25, P75)]	1.050 (1.0,1.1)	1.10 (1.05,1.16)	1210.500	0.030*

Values are expressed as *n* (%) for categorical variables and as median (P25, P75) for continuous variables. LDH, lactate dehydrogenase (U/L); ALB, albumin (g/L); TP, total protein (g/L); INR, international normalized ratio (ratio); Age, years; Height, cm; Weight, kg; Abdominal circumference, cm; Length of hospitalization, days. ICU, Intensive Care Unit. *N*, number. *p < 0.05; ** *p* < 0.01.

### 3.2 Comparison of liver function changes after treatment

Changes in liver function indicators after treatment showed that GGT decreased by 58.5 U/L in the intervention, significantly greater than 7.0 U/L in the control group. ALP decreased by 75.0 U/L in the intervention group, significantly greater than 9.5 U/L in the control group. TBIL decreased by 33.1 μmol/L in the intervention group, significantly greater than 3.85 μmol/L in the control group. DBIL decreased by 9.8 μmol/L in the intervention group, significantly greater than 0.8 μmol/L in the control group. IBIL decreased by 15.8 μmol/L in the intervention group, significantly greater than 1.95 μmol/L in the control group. The differences in the above indicators between the two groups were statistically significant (*p* < 0.01). For other indicators such as AST, ALT, TP, ALB, GLB, and A/G, no statistically significant differences were observed between the two groups (*p* > 0.05) (Table 3).

**Table 3:** Comparison of changes in liver function indicators after treatment.

Indicators	Control group M(P25, P75) (*n* = 80)	Intervention group M(P25, P75) (*n* = 40)	U	*p*
ALT change	−24.500 (−141.5,3.8)	−68.000 (−204.8,6.0)	1343.000	0.152
AST change	−19.500 (−94.3,4.8)	−94.500 (−188.0,7.8)	1350.500	0.165
GGT change	−7.000 (−54.3,8.0)	−58.500 (−153.5,-14.3)	933.000	0.000**
ALP change	−9.500 (−42.0,5.8)	−75.000 (−152.0,-23.5)	777.000	0.000**
TP change	−4.800 (−10.4,1.1)	−4.900 (−9.8,2.1)	1579.500	0.909
ALB change	−2.000 (−4.9,0.2)	−1.350 (−2.9,1.8)	1391.000	0.245
GLB change	−2.200 (−5.5,1.8)	−2.250 (−7.6,1.4)	1406.000	0.280
A/G change	0.000 (−0.1,0.1)	0.025 (−0.1,0.1)	1434.000	0.355
TBIL change	−3.850 (−15.8,1.7)	−33.100 (−105.4,-7.0)	687.500	0.000**
DBIL change	−0.800 (−5.3,0.7)	−9.800 (−62.4,-1.9)	798.000	0.000**
IBIL change	−1.950 (−7.8,1.7)	−15.80 0(−35.2,−2.1)	854.000	0.000**

Values are expressed as median (P25, P75). ALT, alanine aminotransferase (U/L); AST, aspartate aminotransferase (U/L); GGT, gamma-glutamyl transferase (U/L); ALP, alkaline phosphatase (U/L); TP, total protein (g/L); ALB, albumin (g/L); GLB, globulin (g/L); A/G, albumin-to-globulin ratio; TBIL, total bilirubin (μmol/L); DBIL, direct bilirubin (μmol/L); IBIL, indirect bilirubin (μmol/L). *N*, number. *p < 0.05; ** *p* < 0.01.

### 3.3 Comparison of changes in DILI severity grading before and after treatment

Before treatment, significant differences were observed in the distribution of DILI severity grades between the two groups (χ^2^ = 22.323, *p* < 0.01). In the intervention group, 25.00% of patients presented with mild liver injury (Grade 1), which was significantly higher than the 2.50% observed in the control group. Additionally, the proportion of patients with acute liver failure (Grade 4) was higher in the intervention group (32.50%) compared to the control group (18.75%). Conversely, the proportions of patients with moderate (Grade 2) and severe (Grade 3) liver injury in the intervention group were 27.50 and 15.00%, both of which were lower than those in the control group (36.25 and 42.50%). After treatment, patients in the intervention group demonstrated marked improvement in DILI severity. The proportion of patients with mild liver injury (Grade 1) increased to 55.00% in the intervention group, significantly higher than the 2.50% in the control group. Moreover, no cases of acute liver failure (Grade 4) were observed in the intervention group, whereas 3.75% of patients in the control group remained at this severity level. The intergroup difference in DILI severity distribution was more significant after treatment (χ^2^ = 48.543, *p* < 0.01). In terms of treatment response, the remission rate in the intervention group reached 65.00%, compared to 51.25% in the control group; however, this difference did not reach statistical significance (*p* > 0.05) (Table 4). Among non-remission patients, most were clinically stable at the time of discharge. Although some still had persistent biochemical abnormalities, they were transferred to outpatient care for continued treatment and follow-up. Their long-term outcomes (such as chronicity, recurrence, or survival) are being observed and will require further accumulation and analysis of follow-up data.

**Table 4:** Comparison of DILI severity grades and remission rates between the two groups before and after treatment.

Indicators	Grade	Control group [*n* (%)]	Intervention group [*n* (%)]	χ^2^	*p*
DILI Severity (Before Treatment)	Grade 1 (Mild Liver Injury)	2 (2.50)	10 (25.00)	22.323	0.000**
	Grade 2 (Moderate Liver Injury)	29 (36.25)	11 (27.50)		
	Grade 3 (Severe Liver Injury)	34 (42.50)	6 (15.00)		
	Grade 4 (Acute Liver Failure)	15 (18.75)	13 (32.50)		
DILI Severity (After Treatment)	Grade 1 (Mild Liver Injury)	2 (2.50)	22 (55.00)	48.543	0.000**
	Grade 2 (Moderate Liver Injury)	57 (71.25)	10 (25.00)		
	Grade 3 (Severe Liver Injury)	18 (22.50)	8 (20.00)		
	Grade 4 (Acute Liver Failure)	3 (3.75)	0 (0.00)		
Remission Status	Yes	41 (51.25)	26 (65.00)	2.044	0.153
	No	39 (48.75)	1 4(35.00)		

*N*, number. **p < 0.01.

### 3.4 Logistic regression analysis and predictive model construction

To further explore the effects of the intervention and other clinical indicators on the severity of DILI, we constructed an ordinal logistic regression model. Model goodness-of-fit analysis indicated that the final ordinal logistic regression model demonstrated significantly better fit compared to the intercept-only model (χ^2^ = 84.028, df = 7, *p* < 0.01), suggesting satisfactory model performance. Moreover, the Akaike Information Criterion (AIC) and Bayesian Information Criterion (BIC) values decreased to 196.982 and 230.432, supporting an optimal balance between model complexity and information loss (Table 5).

**Table 5:** Likelihood ratio test for the ordinal logistic regression model.

Model	−2 Log Likelihood	χ^2^	*df*	*p*	AIC	BIC
Intercept-only Model	257.010					
Final Model	172.982	84.028	7	0.000	196.982	230.432

df, Degrees of Freedom; AIC, Akaike Information Criterion; BIC, Bayesian Information Criterion.

In the threshold parameters of the model, the regression coefficient corresponding to severe liver injury was 6.001 (*p* < 0.05), indicating that the classification boundary for this grade was statistically significant and could effectively differentiate between varying severities of liver injury. Among all included independent variables, the “treatment Group” (intervention group vs. control group) showed the most significant impact on liver injury severity, with a regression coefficient of −4.004 (*p* < 0.01) and an odds ratio (OR) of 0.018. This suggests that patients in the intervention group had a significantly lower risk of developing higher-grade liver injury, indicating a strong protective effect. In addition, elevated TBIL levels were positively associated with more severe liver injury, with a regression coefficient of 0.015 (*p* < 0.01) and an OR of 1.015. This implies that for every 1 μmol/L increase in TBIL, the risk of progressing to a higher grade of liver injury increased by 1.5%, identifying TBIL as an independent predictive factor. Other variables such as length of hospitalization, age, abdominal circumference, GGT, and ALP did not demonstrate statistical significance in this model (*p* > 0.05), suggesting their limited predictive value for liver injury severity (Table 6).

**Table 6:** Results of the ordinal logistic regression analysis for liver injury severity.

	Indicators	*β*	SE	*p*	OR	OR (95% CI)
Thresholds for Dependent Variable	Grade 1 (Mild Liver Injury)	−2.127	2.219	0.338	8.392	0.108 ~ 649.077
	Grade 2 (Moderate Liver Injury)	1.912	2.234	0.392	0.148	0.002 ~ 11.785
	Grade 3 (Severe Liver Injury)	6.001	2.415	0.013	0.002	0.000 ~ 0.281
Independent Variables	Treatment Group	−4.004	0.619	0.000	0.018	0.005 ~ 0.061
	Length of hospitalization	−0.060	0.041	0.144	0.942	0.870 ~ 1.021
	Age	0.004	0.016	0.790	1.004	0.973 ~ 1.037
	Abdominal Circumference	0.029	0.023	0.209	1.029	0.984 ~ 1.077
	GGT	0.001	0.001	0.483	1.001	0.999 ~ 1.003
	ALP	0.001	0.001	0.601	1.001	0.998 ~ 1.003
	TBIL	0.015	0.003	0.000	1.015	1.009 ~ 1.021

β, Coefficient; SE, Standard Error; OR, Odds Ratio; CI, Confidence Interval; GGT, Gamma-Glutamyl Transferase; ALP, Alkaline Phosphatase; TBIL, Total Bilirubin.

Further analysis of the model’s predictive performance showed an overall accuracy of 75.0%. Notably, the prediction accuracy for moderate liver injury was the highest at 91.05%, followed by mild liver injury at 66.67%. In contrast, the prediction accuracies for severe liver injury and acute liver failure were relatively lower, at 46.15 and 33.33%, respectively. These findings suggest that the model performs well in predicting mild to moderate liver injury, indicating its potential value for clinical application (Table 7).

**Table 7:** Prediction accuracy of the ordinal logistic regression model.

	Actual frequency	Correctly predicted frequency	Prediction accuracy (%)
Grade 1 (Mild Liver Injury)	24	16	66.667%
Grade 2 (Moderate Liver Injury)	67	61	91.045%
Grade 3 (Severe Liver Injury)	26	12	46.154%
Grade 4 (Acute Liver Failure)	3	1	33.333%
Total	120	90	75.000%

To further verify the correlations among key variables, we performed Spearman correlation analysis between the post-treatment liver injury grades and relevant clinical indicators. The results are presented in Table 7. The analysis revealed that the Treatment Group was significantly negatively correlated with liver injury severity (r = −0.412, *p* < 0.01), indicating that patients in the intervention group were more likely to exhibit milder liver injury after treatment. This finding is consistent with the results of the ordinal logistic regression, where the “intervention group” variable was identified as a significant protective factor (OR = 0.018, *p* < 0.01). Additionally, TBIL was positively correlated with liver injury severity (r = 0.380, *p* < 0.01), which aligns with the regression model’s conclusion that TBIL is a significant risk factor (OR = 1.015, *p* < 0.01). Both GGT and ALP showed significant positive correlations with TBIL (r = 0.457 and 0.418, respectively; *p* < 0.01), suggesting a synergistic role of these liver function markers in reflecting liver injury. Other variables, such as length of hospitalization, age, and abdominal circumference, showed no significant correlation with liver injury grade, further supporting their lack of independent predictive value in the logistic regression model (Table 8).

**Table 8:** Spearman correlation matrix of post-treatment liver injury grade and related variables.

Indicators	Mean	SD	Severity grade after treatment	Treatment group	Length of hospitalization	Age	Abdominal circumference	GGT	ALP	TBIL
Severity Grade After Treatment	2.067	0.719	1							
Treatment Group	1.333	0.473	−0.412**	1						
Length of Hospitalization	10.400	5.671	0.074	−0.009	1					
Age	55.692	13.479	−0.145	0.270**	0.052	1				
Abdominal Circumference	80.358	8.990	0.140	−0.144	−0.039	0.003	1			
GGT	203.350	243.604	0.153	0.268**	0.109	0.045	−0.024	1		
ALP	217.558	214.725	0.139	0.268**	0.043	0.153	−0.008	0.583**	1	
TBIL	84.474	105.379	0.380**	0.275**	0.280**	−0.021	−0.057	0.457**	0.418**	1

SD, standard deviation; GGT, Gamma-Glutamyl Transferase; ALP, Alkaline Phosphatase; TBIL, Total Bilirubin. **p < 0.01.

Collinearity diagnostics showed that all variables had variance inflation factor (VIF) values below 10 and tolerance values greater than 0.1, indicating that there was no significant multicollinearity within the model.

## 4 Discussion

Contrary to our initial hypothesis, probiotic intervention did not significantly reduce ALT or AST levels, indicating no direct improvement in hepatocellular injury. However, patients in the intervention group showed significantly greater reductions in cholestatic parameters, including GGT, ALP, TBIL, and its subtypes (DBIL and IBIL), compared with those receiving conventional therapy alone. Furthermore, the degree of improvement in liver injury grading was substantially higher in the intervention group than in the control group. These results suggest that the primary benefits of probiotics in DILI lie in alleviating cholestasis and reducing disease severity, thereby supporting the potential of microbiota-targeted therapy as an adjunctive approach for DILI management.

Previous studies have confirmed that intestinal dysbiosis plays a pivotal role in the pathogenesis and progression of various liver diseases, including MASLD, liver fibrosis, and DILI (13, 14). For instance, Shu et al. reported that probiotic supplementation effectively reduced liver enzyme levels in an animal model of alcoholic liver injury, suggesting the hepatoprotective potential of microbiota modulation (15). Liu et al. further demonstrated that probiotics may ameliorate cholestatic liver injury by regulating bile acid metabolism via the farnesoid X receptor (FXR)–fibroblast growth factor 19 (FGF19) signaling axis (16). In our study, significant reductions in GGT and ALP levels were observed in the intervention group, consistent with the findings of the aforementioned studies. This suggests that probiotics may enhance bile acid metabolism and alleviate hepatobiliary burden, thereby improving cholestatic markers. By contrast, no significant changes were observed in ALT or AST, suggesting that probiotic therapy may exert limited direct effects on hepatocellular injury but predominantly act through cholestasis-related pathways. Moreover, other studies have highlighted the critical role of intestinal barrier dysfunction and microbial imbalance in the pathogenesis of DILI (17). Given the anatomical connection between the gut and liver via the portal vein, microbial metabolites such as lipopolysaccharides (LPS) can activate hepatic Kupffer cells, triggering inflammatory responses through the Toll-like receptor 4 (TLR4)/NF-κB signaling pathway, thereby exacerbating liver injury (18).

This study demonstrated that probiotic intervention significantly improved cholestatic parameters in patients with DILI, particularly with marked reductions in bilirubin, ALP, and GGT levels—biomarkers closely associated with cholestasis. These findings suggest that the hepatoprotective effects of probiotics may be primarily mediated through the regulation of bile acid metabolism and the alleviation of cholestatic injury, rather than through direct reduction of hepatocellular necrosis. Current evidence indicates that probiotics may exert their beneficial effects via multiple synergistic pathways, thereby mitigating hepatic inflammation and dysfunction. Mechanistically, the potential hepatoprotective effects of probiotics may be attributed to the following pathways: (1) Inhibition of LPS-mediated immune activation and inflammation: Probiotics may downregulate the Toll-like receptor 4 (TLR4)/NF-κB signaling pathway, thereby reducing the release of pro-inflammatory cytokines such as tumor necrosis factor-α and interleukin-1β, ultimately alleviating inflammatory liver injury (18, 19). Although inflammatory markers such as CRP were not included in this study, existing evidence suggests that probiotics may exert anti-inflammatory effects through modulation of the TLR4/NF-κB signaling pathway and related cytokine responses. Therefore, the anti-inflammatory explanation in our discussion should be regarded as a potential mechanism supported by previous studies rather than as a direct conclusion from our data. (2) Enhancement of intestinal barrier function: By promoting the expression of tight junction proteins such as occludin and ZO-1, probiotics help reduce intestinal permeability and prevent bacterial translocation and endotoxin leakage, which in turn diminishes the inflammatory load of the portal circulation (20). Previous studies have shown that specific probiotic strains, such as Lactobacillus and Bifidobacterium species, can upregulate the expression of intestinal tight junction proteins, and effectively reduce bacterial translocation and liver inflammatory responses (21, 22). (3) Regulation of bile acid metabolism: Probiotics can activate the FXR/FGF19 signaling axis, promoting the synthesis, transport, and excretion of bile acids, thus alleviating cholestasis and protecting against bile duct injury (16). (4) Promotion of short-chain fatty acid (SCFA) production, such as butyrate: SCFAs exert anti-inflammatory, antioxidant, and hepatoprotective effects by activating receptors such as GPR41 and GPR43 (23). The significant reduction in ALP and GGT levels may reflect alleviation of oxidative stress and structural repair of the biliary system, further supporting the pivotal role of SCFAs in mitigating hepatic oxidative injury (24). These mechanisms are consistent with the observed improvements in cholestatic markers in this study, supporting the multifaceted protective effects of probiotic intervention in DILI. In addition to inflammation resolution, the significant reduction in bilirubin levels may also reflect regulatory effects of probiotics on hepatocellular bile transport proteins, such as restoration of multidrug resistance-associated protein 2 (MRP2) and bile salt export pump (BSEP) expression (25). This hypothesis warrants further investigation at the molecular level to elucidate the precise signaling pathways involved in probiotic-mediated modulation of DILI.

The results also showed that probiotic intervention significantly improved the severity grading of DILI. Compared with the control group, the proportion of patients with mild liver injury (grade 1) in the intervention group increased markedly after treatment (55.0% vs. 2.5%), while the proportions of moderate to severe liver injury and acute liver failure decreased significantly (*p* < 0.01). This improvement is likely associated with multiple mechanisms of probiotics, including modulation of inflammatory responses, optimization of bile acid metabolism, restoration of the intestinal barrier, and reshaping of the gut microbiota (26). Compared with conventional treatment, probiotics may offer additional advantages in promoting bile excretion and reducing hepatocellular stress secondary to cholestasis, thereby facilitating the transition of DILI severity from high-risk to lower-risk states. This finding provides exploratory evidence for the potential role of probiotics in graded intervention for DILI and suggests their promise in supporting improved clinical outcomes and reducing the risk of complications.

In this study, the ordinal logistic regression model demonstrated good fit as confirmed by the likelihood ratio test (*p* < 0.01), with significantly reduced AIC and BIC values, indicating that the included variables had strong explanatory power. Further analysis revealed that the “treatment group” (intervention group vs. control group) was the most significant factor influencing liver injury severity, with an OR of 0.018, suggesting that the intervention significantly reduced the risk of developing more severe liver injury. Additionally, TBIL level was identified as an independent risk factor, with a 1.5% increase in risk for each 1 μmol/L increment (OR = 1.015, *p* < 0.01), highlighting the central role of cholestasis-related biomarkers in predicting DILI severity. The model achieved an overall prediction accuracy of 75.0%, with particularly high performance in predicting moderate liver injury (91.0%), suggesting its potential utility as a supportive tool for early assessment and therapeutic monitoring of DILI severity in clinical practice. Moreover, the model underscored the independent contribution of probiotic intervention to disease severity improvement, providing theoretical support for its standardized application. Future studies may incorporate inflammatory markers, gut-derived metabolites, or imaging parameters to enhance the model’s generalizability and predictive precision. It is noteworthy that although probiotic intervention in this study did not show statistically significant differences in short-term outcomes such as length of hospitalization or ICU treatment, its significant effects on cholestatic marker improvement and severity downgrading suggest a sustained biological impact on liver repair. Furthermore, multivariate logistic regression analysis revealed a significant negative association between probiotic intervention and the risk of severe liver injury, further supporting its independent protective role in the remission process of DILI.

Compared with existing studies, this study provides more robust and comprehensive evidence supporting probiotic intervention in the treatment of DILI on multiple levels. First, the study provided exploratory clinical evidence of probiotics improving cholestatic markers (bilirubin, ALP, GGT) and severity grading in 120 patients, with a relatively large sample size that enhanced statistical power. Second, it systematically compared pre- and post-intervention changes in both hepatobiliary and biochemical parameters and applied a multivariate ordinal logistic regression model to validate the independent protective effect of probiotic therapy, thereby strengthening the basis for causal inference. Third, the study revealed the potential application value of bilirubin, ALP, and GGT in the stratification of DILI severity and efficacy prediction, providing theoretical support for future biomarker development in the context of bile acid metabolism. Finally, unlike most previous studies that have remained at the level of animal experiments or preliminary clinical observations, this study utilized real-world clinical data and systematically assessed the entire intervention–outcome continuum—from probiotic therapy and cholestatic parameter improvement to disease severity downgrading—thus offering initial human evidence and a theoretical foundation for the potential standardized application of probiotics in DILI treatment. Notably, the lack of significant improvement in ALT and AST suggests that probiotic benefits may be more specific to cholestasis-related processes rather than direct amelioration of hepatocellular injury.

Although this study demonstrates improvements over previous research in terms of sample size and statistical methodology, several limitations must be acknowledged. First, as a single-center retrospective study, it may be subject to selection bias, and causal inferences cannot be firmly established. The external generalizability of the findings requires further validation in multicenter, large-scale prospective cohorts. Second, although the sample size was relatively large, there is still room for improvement in subgroup analyses and more detailed exploration of potential effect modifiers. Future studies should adopt more refined patient stratification strategies to enhance interpretability. It is particularly important to note that due to sample size limitations, we only compared the distribution of causative drug categories across groups and did not perform subgroup analyses at the individual drug level; this limitation may have obscured drug-specific effects. Third, although probiotics were prescribed in some patients, this practice reflects local real-world clinical decision-making rather than guideline-based therapy. The absence of large randomized controlled trials investigating probiotics in DILI remains a major limitation, and our findings should be interpreted as exploratory. In addition, all patients in both groups received background supportive therapy with magnesium isoglycyrrhizinate (MgIG) and liver hydrolysate. While MgIG is recommended in the 2023 Chinese Guidelines for the Diagnosis and Treatment of DILI (Grade 1A) and is widely used in China, liver hydrolysate is not guideline-endorsed and represents an empiric supportive agent occasionally used in our center. These center-specific practices may reduce the generalizability of our results to international settings. Moreover, severity grading was based on the Chinese guideline definitions, which differ slightly from Western standards, potentially limiting comparability across studies. Importantly, our initial hypothesis that probiotics would improve transaminases (ALT/AST) was not supported; the observed benefits were confined to cholestasis-related parameters, which may restrict their applicability to hepatocellular-predominant DILI. Furthermore, the lack of long-term follow-up data prevented assessment of sustained benefits such as recurrence, chronicity, or survival. Among non-remission patients, most were clinically stable at discharge but continued to show biochemical abnormalities. They were transferred to outpatient care for treatment and follow-up. Their long-term outcomes are being observed and will require further accumulation and analysis of follow-up data. In addition, inflammatory biomarkers (e.g., CRP, cytokines) were not assessed, which limits our ability to directly validate the proposed anti-inflammatory effects of probiotics; future studies should integrate these markers to clarify the underlying mechanisms. This study also did not include dynamic assessments of key mediating variables such as gut microbiota composition, bile acid profiles, and short-chain fatty acids before and after intervention, which limits empirical support for the gut–liver axis hypothesis. In addition, although LDH was included at baseline to characterize liver injury, follow-up measurements were available only in a small subset of patients, which precluded reliable analysis of treatment-related changes. Future studies should incorporate serial LDH assessments to provide a more comprehensive evaluation of hepatocellular injury. Therefore, the mechanistic pathways by which probiotics may alleviate DILI require systematic validation. Future research should focus on multicenter prospective trials and incorporate multi-omics approaches—including metagenomics, transcriptomics, and metabolomics—to provide mechanistic insights and facilitate clinical translation.

## 5 Conclusion

As an adjunctive strategy for the management of DILI, probiotic intervention significantly improved cholestasis-related parameters (GGT, ALP, TBIL, DBIL, IBIL) and reduced the severity of liver injury, although no significant effects were observed on hepatocellular injury markers (ALT, AST). This real-world study provides novel clinical evidence supporting the potential of microbiota-targeted therapy in DILI, particularly for cholestasis-predominant cases. However, the standardized implementation of probiotics in DILI treatment requires confirmation through multicenter, randomized controlled trials. Future research should focus on defining optimal probiotic strains, dosing regimens, and timing of intervention, and on systematically elucidating the underlying mechanisms using multi-omics approaches to establish a stronger theoretical and clinical foundation for microbiome-based therapy.

## Data Availability

The raw data supporting the conclusions of this article will be made available by the authors, without undue reservation.
